# Anxiolytic and antidepressant profile of the methanolic extract of *Piper nigrum* fruits in beta-amyloid (1–42) rat model of Alzheimer’s disease

**DOI:** 10.1186/s12993-015-0059-7

**Published:** 2015-03-29

**Authors:** Lucian Hritcu, Jaurès A Noumedem, Oana Cioanca, Monica Hancianu, Paula Postu, Marius Mihasan

**Affiliations:** Department of Biology, Alexandru Ioan Cuza University, Bd. Carol I, No.11, Iasi, 700506 Romania; Department of Biochemistry, Faculty of Science, University of Dschang, Dschang, Cameroon; Faculty of Pharmacy, University of Medicine and Pharmacy “Gr. T. Popa”, 16 University Str., Iasi, 700117 Romania

**Keywords:** *Piper nigrum* extract, Beta-amyloid (1–42) peptide, Anxiety, Depression, Oxidative stress, Alzheimer’s disease

## Abstract

**Background:**

*Piper nigrum* L. (Piperaceae) is employed in traditional medicine of many countries as analgesic, antiinflammatory, anticonvulsant, antioxidant, antidepressant and cognitive-enhancing agent. This study was undertaken in order to evaluate the possible anxiolytic, antidepressant and antioxidant properties of the methanolic extract of *Piper nigrum* fruits in beta-amyloid (1–42) rat model of Alzheimer’s disease.

**Methods:**

The anxiolytic- and antidepressant-like effects of the methanolic extract were studied by means of *in vivo* (elevated plus-maze and forced swimming tests) approaches. Also, the antioxidant activity in the amygdala was assessed using superoxide dismutase, glutathione peroxidase and catalase specific activities, the total content of the reduced glutathione, protein carbonyl and malondialdehyde levels. Statistical analyses were performed using one-way analysis of variance (ANOVA). Significant differences were determined by Tukey’s *post hoc* test. *F* values for which p < 0.05 were regarded as statistically significant. Pearson’s correlation coefficient and regression analysis were used in order to evaluate the connection between behavioral measures, the antioxidant defence and lipid peroxidation.

**Results:**

The beta-amyloid (1–42)-treated rats exhibited the following: decrease of the exploratory activity, the percentage of the time spent and the number of entries in the open arm within elevated plus-maze test and decrease of swimming time and increase of immobility time within forced swimming test. Administration of the methanolic extract significantly exhibited anxiolytic- and antidepressant-like effects and also antioxidant potential.

**Conclusions:**

Taken together, our results suggest that the methanolic extract ameliorates beta-amyloid (1–42)-induced anxiety and depression by attenuation of the oxidative stress in the rat amygdala.

## Background

Alzheimer’s disease (AD) is a multifaceted neurodegenerative disorder of the central nervous system characterized by progressive cognitive dysfunction [1,2]. It is known that excessive accumulation of neurotoxic β-amyloid peptide (Aβ) in the brain is the hallmark of AD and the deposition of Aβ causes neuropathological lesions in the brain of patients with AD [3].

These patients often exhibit psychiatric symptoms along with cognitive decline [4]. Emotional symptoms like anxiety and phobia contribute significantly to the clinical profile in mild cognitive impairment (MCI) and AD [5,6]. Emotional behavior critically depends on the amygdala, a region of the temporal lobe that is affected by Aβ and neurofibrillary tangle pathology at early stages of AD [7,8]. Intraneuronal Aβ accumulation in the amygdala enhanced fear and anxiety in AD transgenic mice [9]. Moreover, Aβ accumulation in the basolateral amygdala also contributed to an increase in anxiety in AD transgenic mice [10]. Previously published data suggest that oxidative stress is involved in Aβ (1–42)-induced neurotoxicity and the pathogenesis of AD [11]. It has been shown that the involvement of free radicals in AD includes the presence of elevated levels of protein oxidation, lipid peroxidation products and oxidative damage to mitochondria due to the ability of the Aβ peptide to act as a pro-oxidant. All of these data suggest that the oxidation process clearly unleashes AD [12].

Anxiety and depression increase the severity of cognitive decline in AD patients [4]. Anxiety is more common in individuals with dementia than in individuals without dementia [13] and it is associated with worse quality of life, problem behaviors, limitations in activities of daily living, nighttime awakenings and poorer neuropsychological performance, even after controlling for depression [13]. Depression is one of the most prevalent and life-time threatening forms of mental illnesses, whereas AD is a neurodegenerative disorder that affects more than 37 million people worldwide. Recent studies have highlighted a strong relationship between depression and AD [14].

Dietary supplements have been extensively studied for their beneficial effects on cognition and AD neuropathology and may thus represent a safe, natural treatment for AD [15].

*Piper nigrum L.* (Piperaceae) is one of the most popular spice products in oriental countries (mostly in Southeast Asia) [16]. It has been traditionally used for the treatment of malaria in India and the epilepsy in China [17]. Moreover, the fruits of *P. nigrum* have been widely used for thousands of years in household spices as condiment, as well as for the treatment of cholera and dyspepsia and other gastric ailments and arthritic disorders [18].

Piperine, a major alkaloid of black pepper (*P. nigrum L.*) used extensively as condiment and flavoring for all types of savory dishes [19], also presents analgesic, antiinflammatory, anticonvulsant, antioxidant, antidepressant and cognitive-enhancing effects [20]. It has been shown that piperine protects against neurodegeneration and cognitive impairment in animal model of cognitive deficit like condition of AD [21]. Furthermore, in a recent study we were able to show that the methanolic extract of *P. nigrum* fruits ameliorated Aβ (1–42)-induced spatial memory impairment by attenuation the oxidative stress in the rat hippocampus [2]. Moreover, this may be one of the reasons that this extract could also exert anxiolytic and antidepressant activities.

In this study we examined the effects of the methanolic extract of *P. nigrum* fruits on anxiety and depression levels, as well as the importance of the methanolic extract in oxidative stress status in the amygdala of Aβ (1–42)-treated rats. Correlation between the anxiety-depression-like behaviors and the levels of the main oxidative stress markers from the amygdala of Aβ (1–42)-treated rats, as a result of the methanolic extract administration was also investigated.

## Methods

### Plant material and plant extract

*Piper nigrum* fruits were collected in Dschang, West Region of Cameroon in June 2010 and identified by Victor Nana at the National Herbarium Yaoundé where a voucher specimen was registered and deposited (No. 25818/SFRcam). The air dried and powdered sample (1 kg) was extracted with methanol for 48 h at room temperature. The extract was then filtered and concentrated under reduced pressure to give the crude extract and kept at 4°C until further investigations.

### HPLC (LC/DAD) analysis

To identify the main compounds an Agilent 1200 HPLC system (Agilent Technologies, CA, USA) coupled with DAD detector was used. The working conditions were: Agilent Zorbax Eclipse XDB-C18 column (4.6 × 150 mm, 5 μm); column temperature: 20°C; detection wavelengths: 240, 282, 326, 340 and 521 nm; flow rate: 0.6 mL/min; gradient elution: formic acid 0.1% (solvent A) and acetonitrile (solvent B); the initial conditions were 100% A and 0% B; the gradient program was 5-30-70-90% solvent B at 5-20-30-45 min, after which we switched back to the initial conditions 5 min; sample injection (25 μL of 40 mg/mL dry extract in methanol: water, 7:3).

### Animals

The study used 60 male Wistar rats (3 months old) weighing 250 ± 50 g at the start of the experiment. The animals were housed in a temperature and light-controlled room (22°C, a 12-h cycle starting at 08:00 h) and were fed and allowed to drink water *ad libitum*.

Animals used in this study were treated in accordance with the guidelines of the animal bioethics of the Act on Animal Experimentation and Animal Health and Welfare from Romania and all procedures were in compliance with Directive 2010/63/EU of the European Parliament and of the Council of 22 September 2010 on the protection of animals used for scientific purposes. The protocol was approved by the Committee on the Ethics of Animal Experiments of the Alexandru Ioan Cuza University of Iasi (Permit Number: 1502721). All surgery was performed under sodium pentobarbital anesthesia, and all efforts were made to minimize animal suffering and to reduce the number of animal used.

### Neurosurgery

All surgical procedures were conducted under aseptic conditions, under sodium pentobarbital (50 mg/kg b.w., i.p., Sigma-Aldrich, Germany) anesthesia. Rats were mounted in the stereotaxic apparatus with the nose oriented 11° below horizontal zero plane. Animal model of AD was established by intracerebroventricular (i.c.v.) injection of 400 pmol Aβ (1–42) (beta-amyloid peptide 1–42, Rat, Sigma-Aldrich, Germany), 20 days prior to administration of the methanolic extract according to the procedure established by Laursen and Belknap [22]. Aβ (1–42) was administered right-unilaterally through Hamilton syringe over 4 min, and the syringe was left in place for 5 min after injection before being slowly removed. The injection volume (4 μL) was delivered gradually (1 μL/min) using the following coordinates: 1.5 mm lateral to the midline; 7.4 mm ventral to the surface of the cortex [23]. The sham-operated rats were injected with saline.

### Drug administration

The rats were divided into 6 groups (10 animals per group): (1) Control group (sham-operated) received distilled water treatment; (2) Aβ (1–42) alone-treated group received diazepam (DZP, 1.5 mg/kg) treatment; (3) Aβ (1–42) alone-treated group received tramadol (TRM, 10 mg/kg) treatment; (4) Aβ (1–42) alone-treated group received distilled water treatment; (5) Aβ (1–42)-treated group received 50 mg/kg of the methanolic extract of *P. nigrum* fruits treatment (Aβ (1–42) + P50); (6) Aβ (1–42)-treated group received 100 mg/kg of the methanolic extract of *P. nigrum* fruits treatment (Aβ (1–42) + P100). The methanolic extract of *P. nigrum* fruits was dissolved in distillated water. The administration of the distilled water and the methanolic extract was performed by gastric gavage with biomedical needles (length 7.62 cm, ball diameter 4 mm, straight). The volume administered was 10 mL/kg of body weight, daily, for 21 consecutive days after neurosurgery. Moreover, animals received extract treatment during training in elevated plus-maze and forced swimming tests. Diazepam hydrochloride (Sigma-Aldrich, Germany) and tramadol (Sigma-Aldrich, Germany) were used as positive controls and were injected intraperitoneally (i.p.) in a volume of 1 mL/kg in laboratory rats, 1 h before behaviorally tested. The estimated daily dose of piperine administered to rats was 46 mg for the group received 50 mg/kg of the methanolic extract treatment and 92 mg for the group received 100 mg/kg of the methanolic extract treatment.

### Behavioral testing

#### Elevated plus-maze test

Behavior in the elevated plus-maze (EPM) is also utilized to assess exploration, anxiety, and motor behavior. The EPM consists of four arms, 49 cm long and 10 cm wide, elevated 50 cm above the ground. Two arms were enclosed by walls 30 cm high and the other two arms were exposed. 30 min after the administration of the methanolic extract of *P. nigrum* fruits, each rat was placed in the center of the maze facing one closed arm. Behavior was observed for 5 min., and the time spent and number of entries into the open and enclosed arms was counted [24]. The percentages of time spent in the open arms (time spent in the open arms/time spent in all arms x 100) were calculated. In addition, the total number of open- and enclosed-arm entries (number of crossing), which indicates the exploratory activity of animals [25], was measured. An entry was defined as an animal placing all four paws into an arm, and no time was recorded when the animal was in the central area. The maze floor was cleaned with cotton and 10% ethanol solution between subjects.

#### Forced swimming test (FST)

The FST is the most widely used model for assessing depressive-like response [26]. The depressive-like response was assessed, basically using the same method described by Campos [27], but with modification. On the first day of the experiments (pretest session), rats were individually placed into cylindrical recipients (diameter 30 cm, height 59 cm) containing 25 cm of water at 26 ± 1°C. The animals were left to swim for 15 min before being removed, dried and returned to their cages. The procedure was repeated 24 h later, in a 6 min swim session (test session), 30 min after the administration of the methanolic extract of *P. nigrum* fruits. During the test session, the following behavioral responses were recorded: (1) immobility (time spent floating with the minimal movements to keep the head above the water); and (2) swimming (time spent with active swimming movements).

### Biochemical parameter assay

After behavioral tests, all rats were deeply anesthetized (using sodium pentobarbital, 100 mg/kg b.w., i.p., Sigma-Aldrich, Germany), decapitated and whole brains were removed. Bilateral amygdala was carefully excised. Each of the amygdala samples were weighted and homogenized (1:10) with Potter Homogenizer coupled with Cole-Parmer Servodyne Mixer in ice-cold 0.1 M potassium phosphate buffer (pH 7.4), 1.15% KCl. The homogenate was centrifuged (15 min at 960 × *g*) and the supernatant was used for assays of SOD, GPX, CAT specific activities, total content of reduced GSH, protein carbonyl and MDA levels.

### Determination of SOD activity

The activity of superoxide dismutase (SOD, EC 1.15.1.1) was assayed by monitoring its ability to inhibit the photochemical reduction of nitroblue tetrazolium (NBT). Each 1.5 mL reaction mixture contained 100 mM TRIS/HCl (pH 7.8), 75 mM NBT, 2 μM riboflavin, 6 mM EDTA, and 200 μL of supernatant. Monitoring the increase in absorbance at 560 nm followed the production of blue formazan. One unit of SOD is defined as the quantity required to inhibit the rate of NBT reduction by 50% as previously described by Winterbourn [28]. The enzyme activity is expressed as units/mg protein.

### Determination of GPX activity

Glutathione peroxidase (GPX, E.C. 1.11.1.9) activity was analyzed by a spectrophotometric assay. A reaction mixture consisting of 1 mL of 0.4 M phosphate buffer (pH 7.0) containing 0.4 mM EDTA, 1 mL of 5 mM NaN_3_, 1 mL of 4 mM glutathione (GSH), and 200 μL of supernatant was pre-incubated at 37°C for 5 min. Then 1 mL of 4 mM H_2_O_2_ was added and incubated at 37°C for further 5 min. The excess amount of GSH was quantified by the DTNB method as previously described by Sharma and Gupta [29]. One unit of GPX is defined as the amount of enzyme required to oxidize 1 nmol GSH/min. The enzyme activity is expressed as units/mg protein.

### Determination of CAT activity

Catalase (CAT, EC 1.11.1.6) activity was assayed following the method of Sinha [30]. The reaction mixture consisted of 150 μL phosphate buffer (0.01 M, pH 7.0), 100 μL supernatant. Reaction was started by adding 250 μL H_2_O_2_ 0.16 M, incubated at 37°C for 1 min and reaction was stopped by addition of 1.0 mL of dichromate: acetic acid reagent. The tubes were immediately kept in a boiling water bath for 15 min and the green colour developed during the reaction was read at 570 nm on a spectrophotometer. Control tubes, devoid of enzyme, were also processed in parallel. The enzyme activity is expressed as μmol of H_2_O_2_ consumed/min/mg protein.

### Total content of reduced GSH

Glutathione (GSH) was measured following the method of Fukuzawa and Tokumura [31]. Samples were deproteinized by the addition of 5% end concentration 5-sulfosalicylic acid and centrifugation at 20800 × *g* for 30 min. 200 μL of supernatant was added to 1.1 mL of 0.25 M sodium phosphate buffer (pH 7.4) followed by the addition of 130 μL DTNB 0.04%. Finally, the mixture was brought to a final volume of 1.5 mL with distilled water and absorbance was read in a spectrophotometer at 412 nm and results were expressed as μg GSH/μg protein.

### Determination of protein carbonyl level

The extent of protein oxidation in the amygdala was assessed by measuring the content of protein carbonyl groups, using 2,4-dinitrophenylhydrazine (DNPH) derivatization as described by Oliver [32] and following the indications of Luo and Wehr [33]. Basically, the supernatant fraction was divided into two equal aliquots containing approximately 2 mg of protein each. Both aliquots were precipitated with 10% trichloroacetic acid (TCA, w/v, final concentration). One sample was treated with 2 N HCl, and the other sample was treated with an equal volume of 0.2% (w/v) DNPH in 2 N HCI. Both samples were incubated at 25°C and stirred at 5 min intervals. The samples were then reprecipitated with 10% TCA (final concentration) and subsequently extracted with ethanol-ethyl acetate (1:1, v/v) and then reprecipitated at 10% TCA. The pellets were carefully drained and dissolved in 8 M urea with 20 mM sodium phosphate buffer, pH 6.5. Insoluble debris was removed by centrifugation at 13 000 × *g* at 4°C. The absorbance at 370 nm of the DNPH-treated sample versus the HCl control was recorded, and the results are expressed as nmols of DNPH incorporated/mg of protein based on an average absorptivity of 21.0 mM^−1^ cm^−1^ for most aliphatic hydrazones.

### Determination of MDA level

Malondialdehyde (MDA), which is an indicator of lipid peroxidation, was spectrophotometrically measured by using the thiobarbituric acid assay as previously described by Ohkawa [34]. 200 μL of supernatant was added and briefly mixed with 1 mL of 50% trichloroacetic acid in 0.1 M HCl and 1 mL of 26 mM thiobarbituric acid. After vortex mixing, samples were maintained at 95°C for 20 min. Afterwards, samples were centrifuged at 960 × *g* for 10 min and supernatants were read at 532 nm. A calibration curve was constructed using MDA as standard and the results were expressed as nmol/mg protein.

### Estimation of protein concentration

Estimation of protein was done using a bicinchoninic acid (BCA) protein assay kit (Sigma-Aldrich, Germany). The BCA protein assay is a detergent-compatible formulation based on BCA for the colorimetric detection and quantification of total protein, as previously described by Smith [35].

### DNA fragmentation

Total DNA was isolated from the amygdala samples using the phenol/chloroform method as previously described by Ausubel [36]. 50 mg tissue sample was digested overnight at 37°C in 0.6 mL digestion buffer (100 mM NaCl, 10 mM TRIS/HCl, 25 mM EDTA pH 8.00, 0.5% SDS) containing 0.1 mg/mL proteinase K (Boehringer Mannheim, Germany). The digest was extracted with equal volumes of TRIS-saturated phenol (pH 8.0) (Roti-phenol, Roth, Germany) by shaking gently to completely mix the two phases. The phases were then separated by centrifugation and the aqueous phase (approx. 0.6 mL) was transferred to another tube avoiding interphase. The DNA was then precipitated by adding 300 μL of 7.5 M ammonium acetate (i.e., 1/2 of volume) and equal volume of 100% ethanol at room temperature and shaken gently to mix thoroughly. DNA seen as stringy precipitate was pelleted by centrifugation and washed with 70% ethanol to remove traces of sodium dodecyl sulfate and phenol. After removing ethanol, DNA was air-dried for 10 min at room temperature and suspended with 50 μL of 10 mM TRIS (pH 8.0), 1 mM EDTA. DNA content was determined spectrophotometrically by absorbance at 260 nm and the purity of the DNA was confirmed by a ratio > 1.8 at 260/280 nm. Approximately 0.5 mg genomic DNA was dissolved in a mixture of 10 μL of TRIS-EDTA and 5 μL of gel loading buffer (0.25% bromophenol blue, 0.25% xylene cyanol FF, 30% (v/v) glycerol) and then loaded on a 1.5% agarose gel in TRIS-boric acid-EDTA (TBE) buffer (89 mM Tris boric acid, 2 mM EDTA, pH 8.0). Electrophoresis was performed in TBE at 120 V until sufficient resolution was obtained. A 1-kb DNA ladder (New England Biolabs, Ipswich, MA) was used as a standard size marker. The bands were visualized by ethidium bromide staining under UV light.

### Statistics

Behavioral scores within elevated plus-maze and forced swimming tests and biochemical data were analyzed by one-way analysis of variance (ANOVA) using GraphPad Prism 6 for Windows. All results are expressed as mean + S.E.M. *F* values for which p < 0.05 were regarded as statistically significant. Significant differences were determined by Tukey’s *post hoc* test. Pearson’s correlation coefficient and regression analysis were used in order to evaluate the connection between behavioral measures, the antioxidant defence and lipid peroxidation.

## Results

### Chemical composition of *Piper nigrum* extract

**Figure 1:**
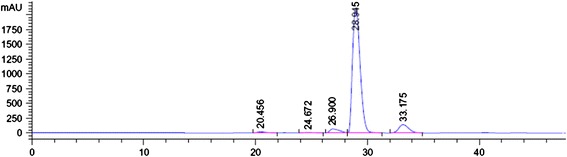
**HPLC data for piperine isolated from the** ***Piper nigrum*** **fruits extract.**

Fourth peak (RT 28.945 min) is represented by piperine, one of the major compounds found in general in pepper species (Figure 1). The purity factor indicates that the peak contains piperine almost 90%. The quantity in our extract is higher than that of other similar amines. The amount of piperine in 1 mg methanolic extract was 0.917 mg. The experiment was in triplicate and the calculated amounts represent the average yield (SD ± 0.0001). Our results are in accordance to literature data for other *Piper* species (*P. nigrum*, *P. guineense* and *P. tuberculatum*) [37].

### Effect of *Piper nigrum* extract on elevated plus-maze behavior

**Figure 2:**
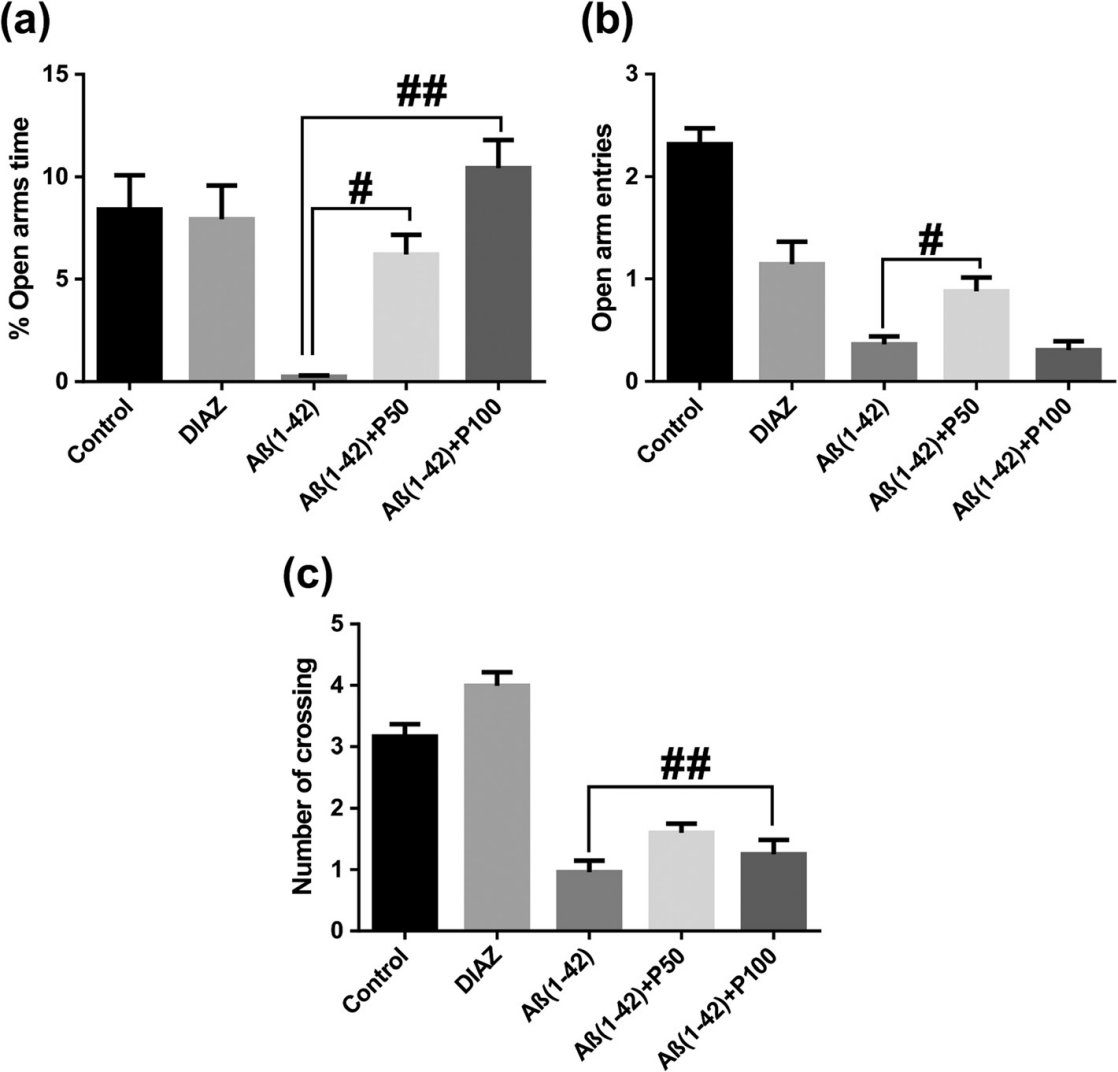
**Effects of the methanolic extract of** ***Piper nigrum*** **fruits (50 and 100 mg/kg) in the elevated plus-maze test on the percentage of the time spent in the open arms (a), the number of open-arm entries (b) and the number of crossing (c) in the Aβ (1–42)-treated rats.** Values are means + S.E.M. (n = 10 animals per group). For Turkey’s *post hoc* analysis - ^#^Aβ (1–42) vs. Aβ (1–42) + P50: p < 0.01 and ^##^Aβ (1–42) vs. Aβ (1–42) + P100: p < 0.0001 **(a)**, ^#^Aβ (1–42) vs. Aβ (1–42) + P50: p < 0.001 **(b)** and ^##^Aβ (1–42) vs. Aβ (1–42) + P100: p < 0.001 **(c)**.

As can be seen in Figure 2a, in the elevated plus-maze task ANOVA revealed a significant overall differences between all groups (F(4,45) = 13.38, p < 0.0001) on the percentage of the time spent in the open arms. Both doses of the methanolic extract, but especially 100 mg/kg, significantly increased the percentage of the time spent in the open arms in Aβ (1–42)-treated groups as compared to Aβ (1–42) alone-treated group. Additionally, Tukey’s *post hoc* analysis revealed a significant difference between Aβ (1–42) vs. Aβ (1–42) + P50 groups (p < 0.01) and Aβ (1–42) vs. Aβ (1–42) + P100 groups (p < 0.0001).

As can be seen in Figure 2b, in the elevated plus-maze task ANOVA revealed a significant overall differences between all groups (F(4,45) = 32.06, p < 0.0001) on the number of open-arm entries. The administration of the methanolic extract, especially 50 mg/kg, significantly increased on the number of open-arm entries of Aβ (1–42)-treated groups as compared to Aβ (1–42) alone-treated group. Additionally, Tukey’s *post hoc* analysis revealed a significant difference between Aβ (1–42) vs. Aβ (1–42) + P50 groups (p < 0.001).

As can be seen in Figure 2c, in the elevated plus-maze task ANOVA revealed a significant overall differences between all groups (F(4,45) = 27.92, p < 0.0001) on the number of crossing (exploratory activity). The administration of the methanolic extract, but especially 100 mg/kg, significantly increased the number of crossing of Aβ (1–42)-treated groups as compared to Aβ(1–42) alone-treated group. Additionally, Tukey’s *post hoc* analysis revealed a significant difference Aβ (1–42) vs. Aβ (1–42) + P100 groups (p < 0.001).

The diazepam treatment significantly increased the percentage of the time spent in the open arms, the number of open-arm entries and the number of crossing as compared to Aβ (1–42)-alone-treated group.

### Effect of *Piper nigrum* extract in the rat forced swimming test

**Figure 3:**
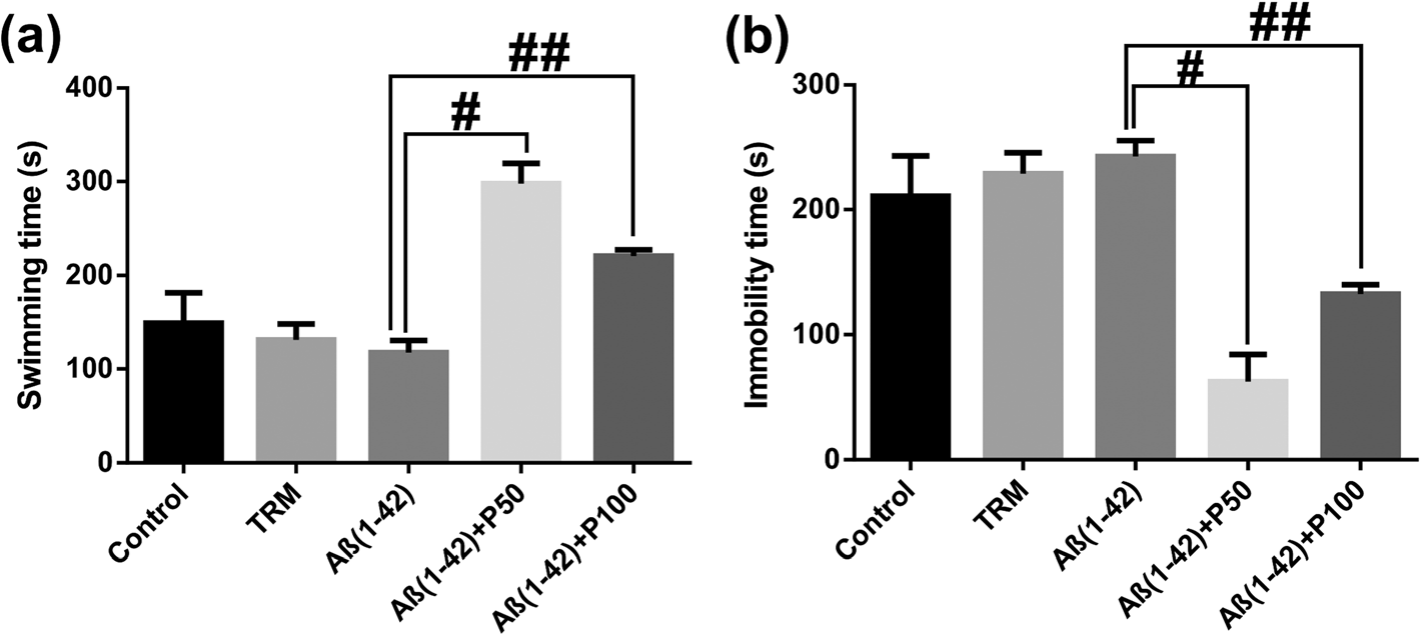
**Effects of the methanolic extract of** ***Piper nigrum*** **fruits (50 and 100 mg/kg) on swimming time (a) and immobility time (b) in the Aβ (1–42)-treated rats during the 6 min period in the forced swimming test.** Values are means + S.E.M. (n = 10 animals per group). For Turkey’s *post hoc* analysis - ^#^Aβ (1–42) vs. Aβ (1–42) + P50: p < 0.0001 and ^##^Aβ (1–42) vs. Aβ (1–42) + P100: p < 0.001 **(a)**, ^#^Aβ (1–42) vs. Aβ (1–42) + P50: p < 0.001 and ^##^Aβ (1–42) vs. Aβ (1–42) + P100: p < 0.01 **(b)**.

In the forced swimming test, ANOVA revealed a significant overall differences between all groups on the swimming time (F(4,45) = 14.07, p < 0.0001) (Figure 3a) and on the immobility time (F(4,45) = 14.33, p < 0.0001) (Figure 3b). Both doses of the methanolic extract, but especially 50 mg/kg, significantly increased swimming time and decreased immobility time of Aβ (1–42)-treated groups as compared to Aβ (1–42) alone-treated group. Additionally, Tukey’s *post hoc* analysis revealed a significant difference between Aβ (1–42) vs. Aβ (1–42) + P50 groups (p < 0.0001) and Aβ (1–42) vs. Aβ (1–42) + P100 groups (p < 0.001) for the swimming time and between the Aβ (1–42) vs. Aβ (1–42) + P50 groups (p < 0.001) and Aβ (1–42) vs. Aβ (1–42) + P100 groups (p < 0.01) for the imobility time.

The tramadol treatment increased the swimming time and decreased the immobility time as compared to Aβ (1–42)-alone-treated group.

### Effect of *Piper nigrum* extract on SOD, GPX and CAT activities

**Figure 4:**
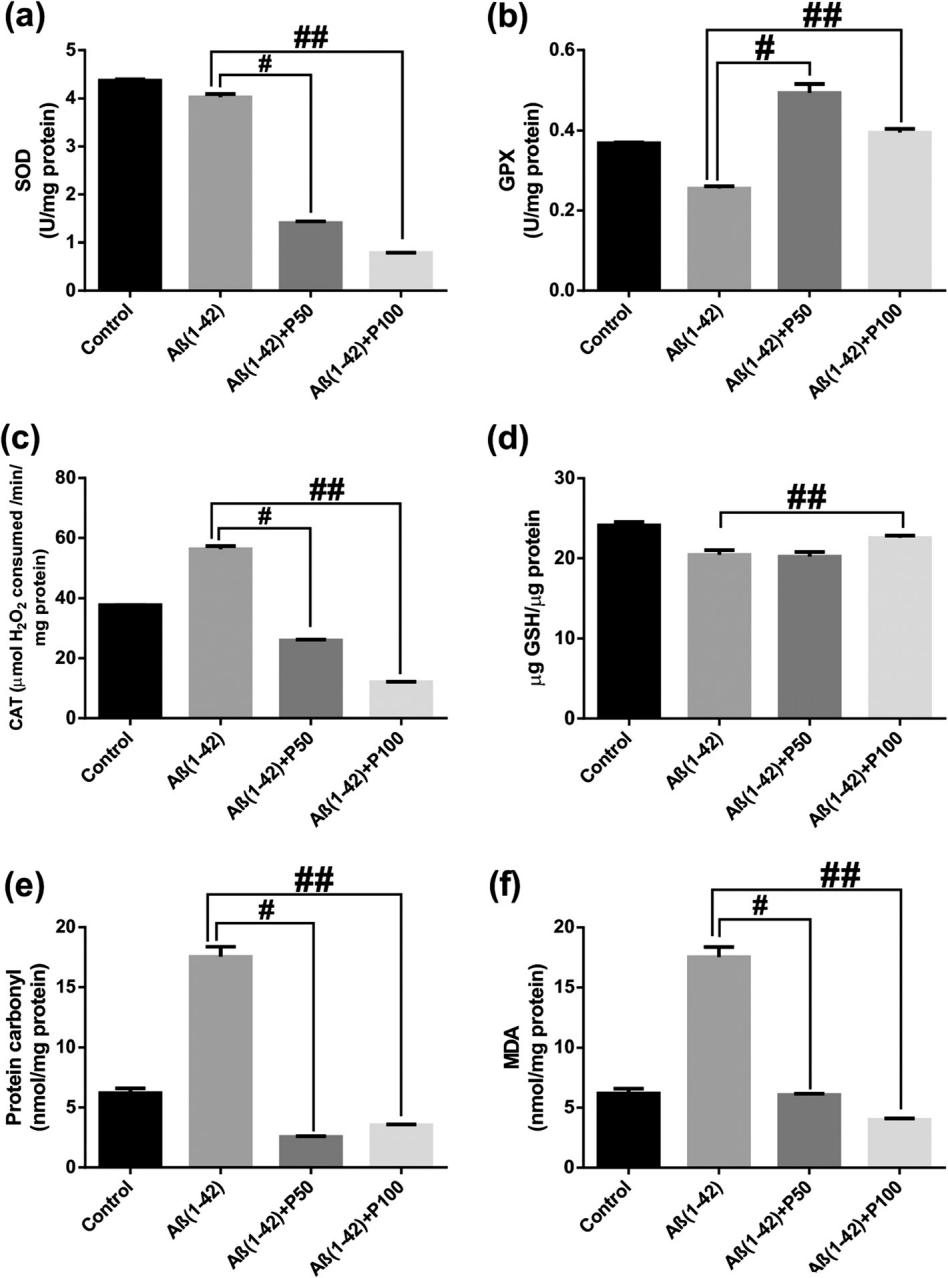
**Effects of the methanolic extract of** ***Piper nigrum*** **fruits (50 and 100 mg/kg) on SOD (a), GPX (b) and CAT (c) specific activities, on total content of reduced GSH (d), protein carbonyl (e) and MDA (f) levels in the Aβ (1–42)-treated rats.** Values are means + S.E.M. (n = 10 animals per group). For Turkey’s *post hoc* analysis - ^#^Aβ (1–42) vs. Aβ (1–42)+P50: p<0.0001 and ^##^Aβ (1–42) vs. Aβ (1–42)+P100: p<0.0001 **(a)**, ^#^Aβ (1–42) vs. Aβ (1–42)+P50: p<0.0001 and ^##^Aβ (1–42) vs. Aβ (1–42)+P100: p<0.0001 **(b)**, ^#^Aβ (1–42) and Aβ (1–42)+P50 groups (p<0.001) and ^##^Aβ (1–42) vs. Aβ (1–42)+P100: p<0.0001**(c)**, ^##^Aβ (1–42) vs. Aβ (1–42)+P100: p<0.01 **(d)**, ^#^Aβ (1–42) vs. Aβ (1–42)+P50: p<0.0001 and ^##^Aβ (1–42) vs. Aβ (1–42)+P100: p<0.0001 **(e)** and ^#^Aβ (1–42) vs. Aβ (1–42)+P50: p<0.0001 and ^##^Aβ (1–42) vs. Aβ (1–42)+P100: p<0.0001 **(f)**.

For SOD, GPX and CAT specific activities estimated in the rat amygdala homogenates, ANOVA revealed a significant overall differences between all groups for SOD (F(3,36) = 20.54, p < 0.0001) (Figure 4a), GPX (F(3, 36) = 66.48, p < 0.0001) (Figure 4b) and CAT (F(3, 36) = 12.77, p < 0.0001) (Figure 4c) specific activities. Both doses of the methanolic extract, but especially 100 mg/kg, significantly decreased the specific activities of SOD, GPX and CAT of Aβ (1–42)-treated groups as compared to Aβ (1–42) alone-treated group. Additionally, Tukey’s *post hoc* analysis revealed significant differences between Aβ (1–42) and Aβ (1–42) + P50 groups (p < 0.0001) and Aβ (1–42) and Aβ (1–42) + P100 groups (p < 0.0001) for SOD specific activity, between Aβ(1–42) and Aβ (1–42) + P50 groups (p < 0.0001) and Aβ (1–42) and Aβ (1–42) + P100 groups (p < 0.0001) for GPX specific activity and between Aβ (1–42) + P50 groups (p < 0.0001), Aβ (1–42) and Aβ (1–42) + P100 groups (p < 0.0001) for CAT specific activity.

### Effect of *Piper nigrum* extract on total content of reduced GSH, protein carbonyl and MDA levels

For the total content of reduced GSH, protein carbonyl and MDA levels estimated in the rat amygdala homogenates, ANOVA revealed significant overall differences between all groups for reduced GSH content (F(3, 36) = 14.64, p < 0.0001) (Figure 4d), protein carbonyl level (F(3, 36) = 22.44, p < 0.00001) (Figure 4e) and MDA level (F(3, 36) = 17.49, p < 0.0001) (Figure 4f). Both doses of the methanolic extract, but especially 100 mg/kg, significantly increased the total content of reduced GSH and MDA level of Aβ (1–42)-treated groups as compared to Aβ(1–42) alone-treated group. Also, the administration of the methanolic extract (50 mg/kg) in Aβ (1–42)-treated group significantly decreased protein carbonyl level of Aβ (1–42)-treated group as compared to Aβ (1–42) alone-treated group. Additionally, Tukey’s *post hoc* analysis revealed significant differences between Aβ (1–42) and Aβ (1–42) + P100 groups (p < 0.01) for the total content of reduced GSH, between Aβ (1–42) and Aβ (1–42) + P50 groups (p < 0.0001) and Aβ (1–42) and Aβ (1–42) + P100 groups (p < 0.0001) for protein carbonyl level and between Aβ (1–42) and Aβ (1–42) + P50 groups (p < 0.0001) and Aβ (1–42) and Aβ(1–42) + P100 groups (p < 0.0001) for MDA level.

These results support the hypothesis that the methanolic extract of *P. nigrum* fruits may have induced a decrease in neuronal oxidative stress.

**Figure 5:**
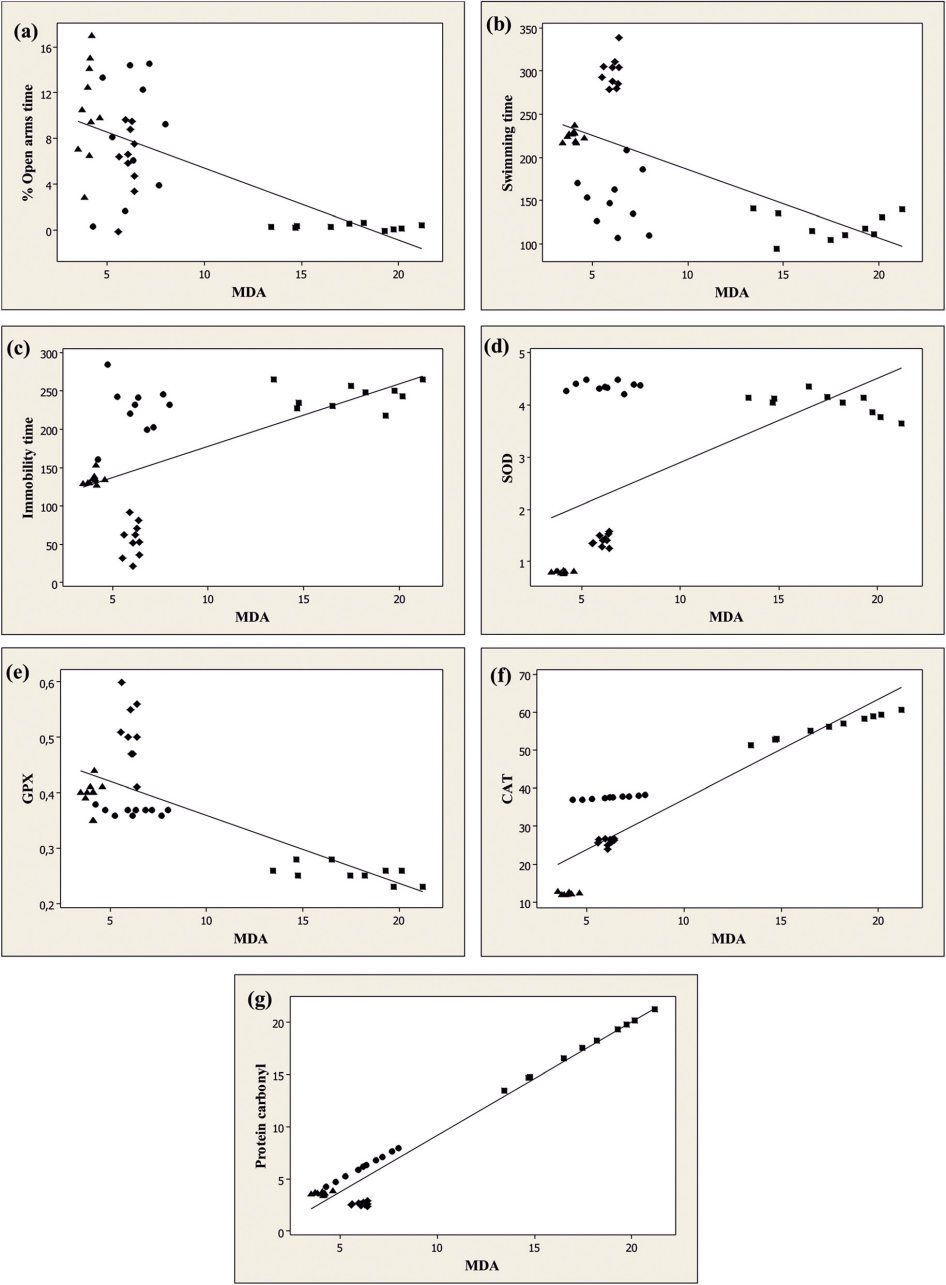
**Pearson’s correlation between the percentage of the time spent in the open arms vs. MDA (a), swimming time vs. MDA (b), immobility time vs. MDA (c), SOD vs. MDA (d), GPX vs. MDA (e), CAT vs. MDA (f) and protein carbonyl vs. MDA (g) in control group (●), Aβ (1–42) alone treated-group (■), Aβ (1–42) + P50 group (♦) and Aβ (1–42) + P100 group (▲).**

More importantly, when linear regression was determined, significant correlations between the percentage of the time spent in the open arms vs. MDA (n = 40, r = −0.662, p < 0.0001) (Figure 5a), swimming time vs. MDA (n = 40, r = −0.608, p < 0.0001) (Figure 5b) and immobility time vs. MDA (n = 40, r = 0.571, p < 0.0001) (Figure 5c) were evidenced. Additionally, a significant correlation was evidenced by determination of the linear regression between SOD vs. MDA (n = 40, r = 0.565, p < 0.0001) (Figure 5d), GPX vs. MDA (n = 40, r = −0.728, p < 0.0001) (Figure 5e), CAT vs. MDA (n = 40, r = 0.890, p < 0.0001) (Figure 5f) and protein carbonyl vs. MDA (n = 40, r = 0.973, p < 0.0001) (Figure 5g).

These data suggest that anxiolytic and antidepressant responses within elevated plus-maze and forced swimming test and increase of the antioxidant defence along with decrease of lipid peroxidation and protein oxidation could related with the involvement of the methanolic extract of *P. nigrum* fruits in neuroprotection against Aβ (1–42)-induced neuronal oxidative stress generation.

### Effect of *Piper nigrum* extract on DNA fragmentation

**Figure 6:**
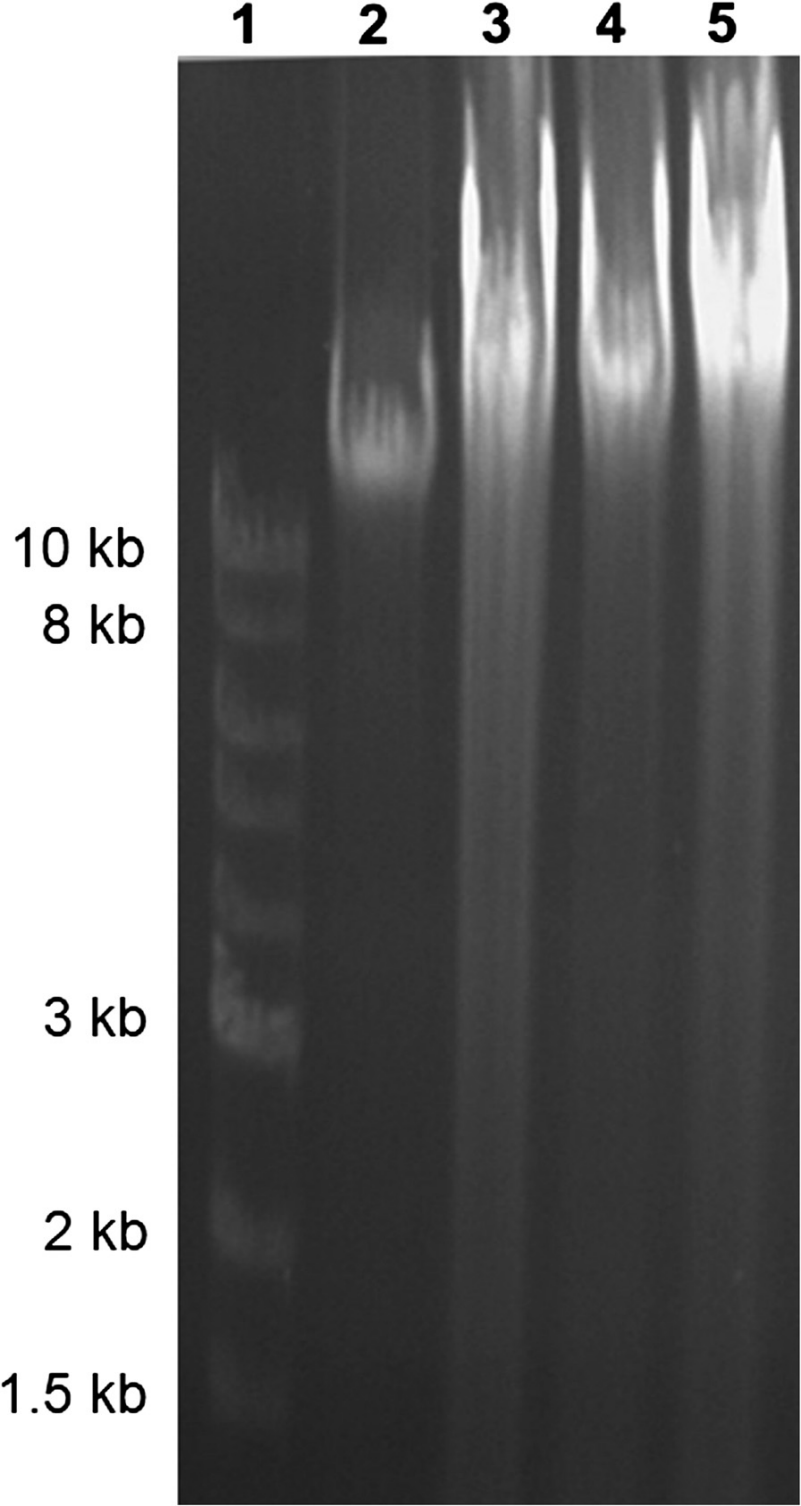
**Effects of the methanolic extract of** ***Piper nigrum*** **fruits (50 and 100 mg/kg) on DNA fragmentation by agarose (1.5%) gel electrophoresis.** Lane 1: DNA ladder; lane 2: control group; lane 3: Aβ (1–42) alone-treated group; lane 4: Aβ (1–42) + P50 group and lane 5: Aβ (1–42) + P100 group.

In our study, DNA cleavage patterns were absent in the methanolic extract groups (Figure 6), suggesting that the methanolic extract of *P. nigrum* fruits protects against neurotoxicity and this effect could be related to its antioxidant activity.

## Discussion

The present study was aimed to examine the anxiety-like behavior and depressive-like response following administration of the methanolic extract of *P. nigrum* fruits rats in subjected to i.c.v. injection of Aβ (1–42). Consequently, i.c.v. injection of Aβ (1–42) causes anxiety-like behavior and depressive-like response, in accordance with previous investigations [9,10].

The HPLC analysis indicated piperine as the main component of our methanolic extract suggesting that this could be the main constituent responsible for the observed anxiolytic and antidepressant effects in Aβ (1–42)-treated rats. It has been reported that piperine inhibited monoamine oxidase activity, increased monoamine neurotransmitters levels, and thus produced antidepressant-like activity in various mouse models of behavioral despair [38]. The antidepressive effect of piperine has been also observed in mice exposed to chronic mild stress and it was linked to up-regulation of hippocampal progenitor cell proliferation [19]. This antidepressant-like effect of piperine in chronically stressed mice was also shown to be mediated by brain-derived neurotrophic factor signaling [39].

Neuropsychiatric symptoms appear in AD patients which range from abnormal motor behavior, depression and anxiety and personality alterations such as aggression and irritability [40]. The elevated plus-maze is recognized as a valuable model able to predict anxiolytic- or anxiogenic-like effects of drugs in rodents [41].

As shown in Figure 2a and Figure 2b, the percentage of the time spent in the open arms and the number of open-arm entries of Aβ (1–42)-treated rats were significantly decreased. This indicates that the Aβ (1–42)-treated rats experienced high levels of anxiety and were suitable for evaluating the presumed anxiolytic substances [24]. Furthermore, after the administration of the methanolic extract, the time spent in the open arms of Aβ (1–42)-treated rats significantly increased in a dose-dependent manner. The number of open-arm entries of Aβ (1–42)-treated rats increased in the Aβ (1–42) + P50 group as compared to the Aβ (1–42)-alone treated group. Additionally, the exploratory activity of Aβ (1–42)-treated rats as evidenced by number of crossing (Figure 2c) significantly increased after administration of the methanolic extract, especially in the Aβ (1–42) + P100 group as compared to the Aβ (1–42)-alone treated group. These results are strengthened by the fact that the benzodiazepine DZP, well-known as positive standard anxiolytic [42], was used as positive control comparatively to the methanolic extract in all of our experimental conditions.

The anxiety indicators in the elevated plus-maze (the percentage of the time spent in the open arms and the number of open-arm entries) showed up being sensitive to the agents which were thought to act via the GABA_A_ receptor complex [43]. The therapeutic action of the benzodiazepines and other pharmacological compounds used to treat anxiety, panic, insomnia, and epilepsy is mediated by an enhancement of GABAergic neuronal inhibition through GABA_A_ receptors [44,45]. Moreover, it is reported that piperine modulates γ-aminobutyric acid (GABA) type A (GABA_A_) receptors and exhibited antidepressant activity in rodents [46,47]. In light with these reports, our high-piperine containing methanolic extract have increased anxiolytic-like behavior and exploratory activity in Aβ (1–42)-treated rats.

The forced swimming test has been validated as a suitable tool for predicting the antidepressant properties of drugs [48]. When rodents are forced to swim in a confined space, after an initial period of struggling, they would become immobile, resembling a state of despair and mental depression. This inescapable stressful situation can be evaluated by assessing different behavioral strategies [49].

As shown in Figure 3a and Figure 3b, the swimming time decreased and the immobility time increased in Aβ (1–42) alone-treated group as compared to control. This indicates that the Aβ (1–42)-treated rats experienced more depressive-like behavior. Next, after being given the methanolic extract, orally for 21 consecutive days, the swimming time significantly increased, especially in the Aβ (1–42) + P50 group as compared to the Aβ (1–42) alone-treated group. We also observed a significant decrease in the immobility time of the methanolic extract treated rats, especially in Aβ (1–42) + P50 group, as compared to Aβ (1–42) alone-treated group.

These results suggest an antidepressant-like effect of the methanolic extract in response to an inescapable stress. We used tramadol as a positive control in the methanolic extract assay for all of our experimental conditions. Tramadol has been studied in the forced swimming test in rats, a test developed to predict the antidepressant action of drugs [50].

Oxidative stress is also involved in the mechanism of Aβ-induced neurotoxicity [51,52] and AD pathogenesis [51]. Exposure to Aβ increased lipid peroxidation, protein oxidation and the formation of hydrogen peroxide in cultured cells [53]. Similarly, the increases in lipid peroxidation, protein carbonyl and oxidation of mitochondrial DNA have been observed in the brains of AD patients [54]. Analysis of AD brains demonstrates an increase in lipid peroxidation products in the amygdala, hippocampus and parahippocampal gyrus of the AD brain compared with age-matched controls [55]. Furthermore, it has been reported that many neuropsychiatric/neurodegenerative disorders are associated with dysregulation of the hypothalamic-pituitary-adrenal (HPA) axis, as well as with oxidative stress, it is possible that oxidative stress disturbs the HPA-axis function, altering the level of corticosterone with subsequent effects on hypothalamus and amygdala [56]. Also, other reports have shown that increased oxidative stress in the hypothalamus and amygdala can alter the function of fear-related circuits in the brain. This may play an important role in behavioral regulation and development of anxiety [57,58].

In our study, Aβ (1–42) alone-treated rats exhibited increase of CAT specific activity, elevated protein carbonyl and MDA levels and decrease of the total content of reduced GSH and GPX specific activity in the amygdala homogenates. Additionally, the specific activity of SOD in Aβ(1–42) alone-treated rats exhibited a small decrease as compared to control rats. The enzymes catalase and SOD are the major defenses against reactive oxygen species (ROS). SOD converts superoxide anions into H_2_O_2_, and catalase converts H_2_O_2_ to molecular oxygen and water. SOD exists in two forms: Cu/ZnSOD is present primarily in the cytoplasm while MnSOD is present primarily in the mitochondria [59]. Aβ is a metalloprotein that displays a high affinity binding of Cu^2+^, Zn^2+^ and Fe^3+^ ions [60]. However, since the formation of amyloid plaques is associated with metal-mediated Aβ aggregation [61], a possible explanation for the observed decrease of SOD activity could be attributed to the perturbation of the brain metals levels [62]. The brain is a specialized organ that concentrates metal ions and changes in brain metals have been implicated in several neurodegenerative disorders such as AD [63]. Moreover, since the oxidative stress significantly increased in Aβ (1–42) alone-treated rats, consequently the catalase activity intensified. The increase of the CAT specific activities showed to correspond with the increase in the protein carbonyl and MDA levels in the amygdala homogenates suggesting that these events are needed to scavenge superoxide radicals induced by Aβ (1–42). It has been suggested that oxidative damage to proteins and DNA in addition to lipids is an important early event in the pathogenesis of AD [64]. ROS-mediated reactions with proteins lead to the formation of protein carbonyl derivative, which serves as a marker of ROS-mediated protein damage. With this marker, it was established that oxidative-damaged protein is associated with aging and AD [65]. The increased protein carbonyl in the AD brain, together with the altered activities of antioxidant enzymes (in some cases due to oxidation), coupled with studies showing that Aβ (1–42)-induced neuronal protein oxidation can be inhibited by antioxidants [66], indicate that Aβ-induced protein oxidation may account, in part, for neurodegeneration in the AD brain. MDA level, an index of lipid peroxidation produced by free radicals, it is the intermediate product of lipid pigment formation process. It can indirectly reflect the lipid peroxidation level, leading to nerve cells damage within the protein metabolism disorder caused by cell dysfunction [67]. Consistently, increased lipid peroxidation was observed in an animal model of Alzheimer amyloidosis [68]. Moreover, both doses of the methanolic extract 50 and 100 mg/kg (but especially 100 mg/kg) with high amount of piperine, restored the specific activities of SOD (Figure 4a) and CAT (Figure 4c) and only 50 mg/kg increased the GPX activity (Figure 4b) in the amygdala homogenates of Aβ (1–42)-treated rats. As expected for the antioxidant agents, both doses of the methanolic extract (50 and 100 mg/kg), decreased the protein carbonyl (Figure 4e) and MDA (Figure 4f) levels along with the increase of the total content of reduced GSH (Figure 4d) in the amygdala homogenates of Aβ (1–42)-treated rats.

Our study support the hypothesis where decreased SOD and CAT specific activities in Aβ (1–42)-treated rats treated with the methanolic extract can lead to decreased production of intracellular of H_2_O_2_ with a simultaneous increase of GPX activity. This could decrease the stimulation of lipid peroxidation and protein oxidation, implying that the methanolic extract possesses strong antioxidant profile.

Additionally, when linear regression was determined, a significant correlation between the percentage of the time spent in the open arms vs. MDA, swimming time vs. MDA, immobility time vs. MDA, SOD vs. MDA, GPX vs. MDA, CAT vs. MDA and protein carbonyl vs. MDA was observed. These results may suggest that the increase of anxiolytic and antidepressant responses within elevated plus maze and forced swimming test and the antioxidant defence along with decrease of lipid peroxidation and protein oxidation could be correlated with involvement of the methanolic extract in neuroprotection against Aβ (1–42)-induced oxidative stress generation in the rat amygdala. Also, we reported the absence of DNA cleavage patterns in the amygdala of the Aβ (1–42)-treated rats treated with the methanolic extract, suggesting that the methanolic extract possesses neuroprotective and antiapoptotic activities.

## Conclusions

In summary, the methanolic extract of *P. nigrum* fruits has anxiolytic and antidepressant effects, and may confer neuroprotection due to alleviation of oxidative stress induced by Aβ (1–42) injection in the rat amygdala. Furthermore, administration of the methanolic extract might offer a useful alternative or complementary choice in either the prevention or the treatment of psychiatric condition close related to AD conditions.
